# A perspective on neuroethology: what the past teaches us about the future of neuroethology

**DOI:** 10.1007/s00359-024-01695-5

**Published:** 2024-02-27

**Authors:** M. Jerome Beetz

**Affiliations:** Zoology II, Biocenter, University of Würzburg, 97074 Würzburg, Germany

**Keywords:** Neuroethology, Comparative physiology, Animal behavior, Electrophysiology

## Abstract

For 100 years, the Journal of Comparative Physiology-A has significantly supported research in the field of neuroethology. The celebration of the journal’s centennial is a great time point to appreciate the recent progress in neuroethology and to discuss possible avenues of the field. Animal behavior is the main source of inspiration for neuroethologists. This is illustrated by the huge diversity of investigated behaviors and species. To explain behavior at a mechanistic level, neuroethologists combine neuroscientific approaches with sophisticated behavioral analysis. The rapid technological progress in neuroscience makes neuroethology a highly dynamic and exciting field of research. To summarize the recent scientific progress in neuroethology, I went through all abstracts of the last six International Congresses for Neuroethology (ICNs 2010–2022) and categorized them based on the sensory modalities, experimental model species, and research topics. This highlights the diversity of neuroethology and gives us a perspective on the field’s scientific future. At the end, I highlight three research topics that may, among others, influence the future of neuroethology. I hope that sharing my roots may inspire other scientists to follow neuroethological approaches.

**Supplementary Information:**

The online version contains supplementary material available at 10.1007/s00359-024-01695-5.

## Introduction

The neural mechanisms of behavior represent the central pillar of neuroethology, a discipline pioneered by Karl von Frisch, Erich von Holst and Theodore Bullock in the late 1950s. In contrast to systems neuroscience, where a reductionist approach under well controlled conditions is desired, neuroethologists seek to conduct experiments in a more naturalistic context (Miller et al. 2022; Hoffmann et al. 2023). This includes monitoring brain activity in behaving animals (Yartsev and Ulanovsky 2013; Martin et al. 2015; Seelig and Jayaraman 2015; Vinepinsky et al. 2017; Jin et al. 2020; Beetz et al. 2022; Perentos et al. 2022; Wosnitza et al. 2022; Agarwal et al. 2023; Gutnick et al. 2023; Wallach and Sawtell 2023) and conducting the recordings in the field (Hoffmann et al. 2019; Eliav et al. 2021; Römer 2021). Rapid technological progress makes the field of neuroethology unpredictable, but at the same time highly dynamic. The interests of neuroethologists are represented by the International Society for Neuroethology (ISN), which organizes the International Congress on Neuroethology (ICN) that takes place every 2 years. To get an idea of the scientific future of neuroethology, I reflect on the recent directions of the field by going through all ICN contributions of the last 13 years and categorizing them into sensory modalities, taxa, and scientific topics. After reviewing the recent past, I will share my perspective on the future of neuroethology. To this end, I will highlight three research topics that recently fascinated me and that may inspire many scientists. To get an overview on the current funding situation or political and infrastructural challenges that many neuroethologists face in different countries, I recommend recently published perspectives (Silva et al. 2022; Zupanc and Rössler 2022; Tomsic and Silva 2023).

Box 1: My neuroethological roots As a trained neuroscientist who started recording from visual neurons of desert locusts in a dark chamber, far away from any desert, I was always keen to discuss the behavioral relevance of my scientific findings (Beetz et al. 2016c). To this end, I joined the ISN and attended my first ICN in Uruguay in 2016. There, I was tremendously inspired to further investigate the loop between nervous systems and behavior. At the end of my master studies, I was still puzzled—what can neural recordings in restrained animals while presenting artificial stimuli tell me about how the brain operates in a behaving animal. This question has set my research path from the beginning of my PhD. As a PhD student, I shifted my focus from invertebrates to vertebrates and conducted neural recordings in echolocating bats. For short-range orientation, bats extract spatial information from echoes (Beetz and Hechavarría 2022). Given that the behavioral relevance of the echolocation signals is clear, I tested how such signals are processed in the bat brain (Beetz et al. 2016a, b; 2017). To mimic a naturalistic stimulus scenario, I presented echolocation signals in the presence of ambient noise that interfered with the echolocation signals (Beetz et al. 2018). After investigating how the stimulus context affects neural processing, I wanted to gain insight into the influence of behavior on neural processing. To this end, I started my postdoctoral research with the goal of monitoring brain activity from flying monarch butterflies tethered in a flight stimulator. While developing neural recordings from tethered flying butterflies, I noticed that the animal’s state of locomotion, i.e., quiescence, or flying, substantially affected the spatial tuning of compass cells (Beetz et al. 2022). These findings demonstrate that my initial goal to monitor brain activity from a behaving animal was essential to understand the interactions between brain and behavior. However, this does not mean that experiments conducted in restrained animals are worthless. Under restrained conditions, neural mechanisms and circuits can often be better characterized than in the field. In 2022, I started my own research group that investigates the spatial memory of insects (Konnerth et al. 2023).

## Definition of neuroethology

While most scientists may agree that neuroethology investigates the neural mechanisms of behavior, most neuroethologists would emphasize the relevance of investigating ‘natural behavior’ (Miller et al. 2022). This is not surprising because ethology, the study of natural behavior, represents an essential root of neuroethology. But what defines natural behavior? Is it restricted to innate behavior, or does it also include learned behavior? What about a behavior learned in captivity that would not be observed in the wild? Are such behaviors beyond the scope of neuroethology? This had been debated in the early years of the field. Graham Hoyle, another pioneer of neuroethology supported a rather restricted view of neuroethology. He feared that the field would otherwise ‘expand into a diffuse vapor without any substance at all’ (Hoyle 1984). Hoyle shared the opinion that ‘most complex behaviors […] fall into the category of instinctive acts. They require no experience of the behavior in its context, nor learning, for their perfect execution.’ (Hoyle 1984). In contrast, Theodore Bullock, the first ISN president, defined neuroethology under a much broader scope: ‘Neuroethology is used when there is some emphasis upon or relevance to the understanding of natural behavior’ (Bullock 1990). This also implies that even reduced or artificial approaches are eligible as long as they aim to elucidate the neural mechanisms underlying natural behavior. One prime example who followed a reductionist approach in his early career was Eric Kandel, who was awarded with the Nobel Prize in 2000. Instead of working with billions of neurons in a rat brain, he focused first on a relatively simple, in terms of number of neurons, nervous system. To this end, he investigated the gill-withdrawal response of the sea slug *Aplysia* to understand the neural principles of learning and memory. Because his findings on gastropods were not fundamentally different from the cellular mechanisms that were described later in mammals, Kandel’s work is not only relevant for marine biologists with a keen interest in sea slugs but also for the entire neuroscience community. His reductionist approach of working on invertebrates before shifting his attention to vertebrates undoubtedly inspires me.

## Representation of sensory modalities and the fascination for ‘hidden modalities’

**Fig. 1:**
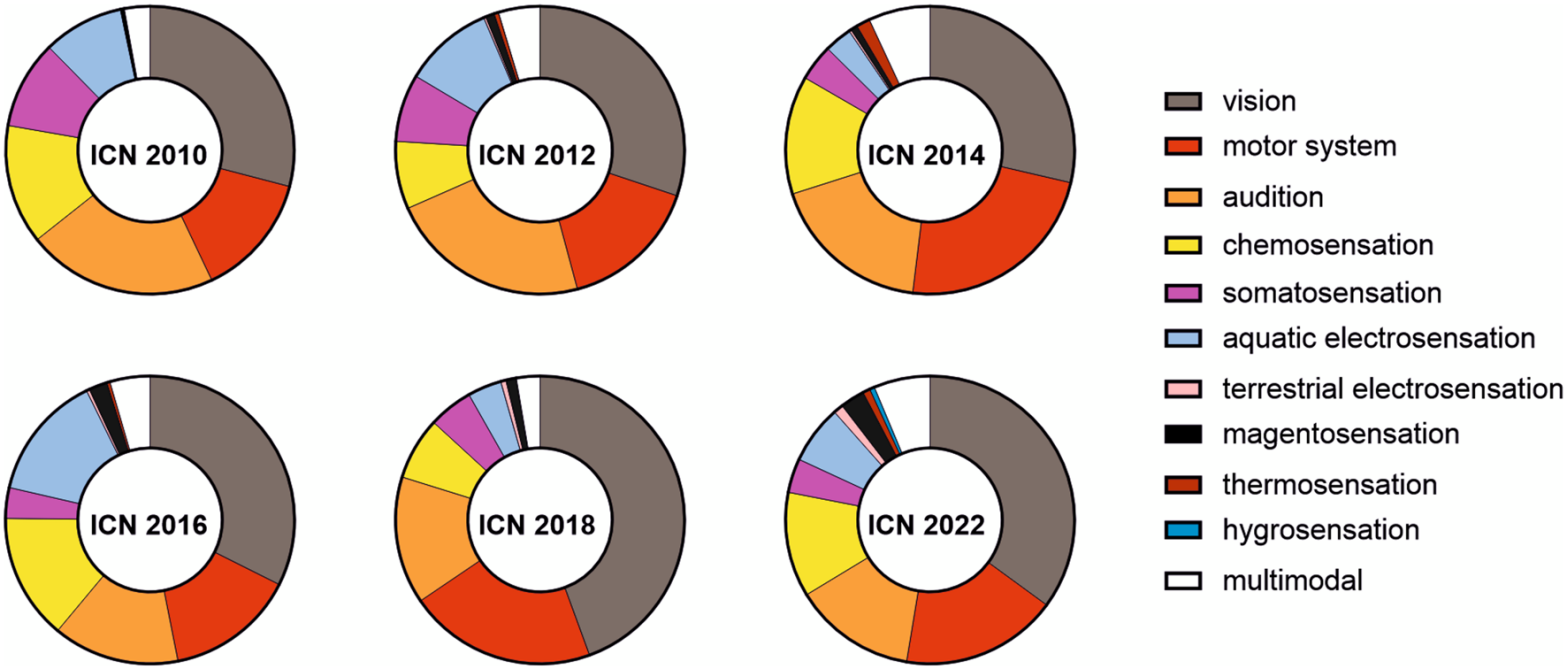
Proportion of sensory modalities and motor system represented at the last six ICNs. Values are listed in Table S1

To get an idea of the scientific future of neuroethology, I first want to review the recent progress in the field. To this end, I categorized 2636 abstracts from the last six ICNs (2010–2022) into the sensory modalities. Given the behavioral relevance of vision, it is not surprising that 28% of the ICN contributions focused on the visual sense (Fig. 1, Table S1). In addition, the diversity of eye designs across the animal kingdom further triggers the fascination for vision (Sumner-Rooney 2023; Warrant 2023). The second most represented sensory modality was the auditory sense representing with about 15% of abstracts. During the spring, one of the first sensory perceptions of the day is a singing bird, an auditory experience that gets replaced by chorusing frogs or crickets in the early evening hours. Hence, acoustic communication in diverse species is central to the research of many neuroethologists (Kelley 2004; Elie and Theunissen 2020; Römer 2021; Narins et al. 2023). However, the acoustic sense is also useful for nocturnal orientation, whether it is used in the context of echolocation in bats (Beetz and Hechavarría 2022) or localizing potential prey by passively listening to prey-emitted sounds (Singheiser et al. 2012; Brewton et al. 2018). During foraging, chemosensation is also essential to localizing food and determining its quality. About, 9% of the ICN contributions were about chemosensation, which includes olfaction and gustation. With about 5% of the contributions, somatosensation (including nociception) was represented by a relatively low number of contributions at the ISN. However, understanding how sensory stimuli are neuronally processed is not sufficient to explain behavior. We must also understand how motor systems operate. Given its importance to behavior, motor systems were central to about 15% of the ICN contributions (Fig. 1, Table S1). Motor systems are not only important for limb control, but also in the context of digestion. For example, research on the stomatogastric nervous system has a long tradition in neuroethology (Marder and Bucher 2007; Daur et al. 2016).

Aside from the ‘classic’ senses, many neuroethologists are fascinated by sensory modalities that go beyond our own perception. For example, electric fish with their active electrosensation represent about 7% of the ICN contributions (‘aquatic electrosensation’, Fig. 1, Table S1). In 2013, the discovery that even terrestrial animals such as bees perceive electric fields (Clarke et al. 2013; Greggers et al. 2013) was the beginning of a new research direction in neuroethology. This gets reflected by an increase in ICN contributions on terrestrial electrosensation from 0.2 to 1%, over the last 10 years. Since then, terrestrial electrosensation has been described in many other arthropods (England and Robert 2022). Spiders use electrostatic forces for ballooning, a strategy for aerial dispersal (Morley and Robert 2018) and more recently it has been demonstrated that ticks are passively attracted by electrostatic fields of their hosts (England et al. 2023). The more we discover about terrestrial electrosensation, the more it seems to be that electrosensation is ecologically and evolutionarily more diverse than initially thought. Future studies might focus on the sensory processing of terrestrial electrosensation and to compare this with aquatic electrosensation.

Another sensory modality that humans cannot perceive is magnetosensation. Many animals, including vertebrates and invertebrates, detect and use the earth’s magnetic field for orientation (Mouritsen 2018). At first glance, too weak to be detected by receptors and too sensitive for electromagnetic disturbances, the behavioral relevance of the earth’s magnetic field has often been questioned. However, behavioral studies convincingly demonstrate that many organisms indeed use the earth’s magnetic field for orientation (Lohmann et al. 2022; Wiltschko and Wiltschko 2022). Aside from the behavioral evidence, physiological processes underlying magnetosensation are less clear (Putman 2022). The most promising theory, the ‘radical-pair’ theory, hypothesizes the involvement of light and quantum physics in the process (Hore and Mouritsen 2016). However, magnetoreceptors have so far not been convincingly described, and we are even further away from dissecting the neural circuit of magnetosensation in any taxon.

Although many nervous systems are biased to invest energy to process certain sensory modalities, behavior is often the result of multimodal processing. For instance, an echolocating bat with its highly specialized auditory system additionally uses visual information for orientation (Boonman et al. 2013). Bats may also use a magnetic compass to cover distances that are too long for acoustic or visual orientation (Holland et al. 2010; Schneider et al. 2023). Electric fish represent another animal group that has been initially thought to rely mostly on their active electric sense for orientation (Landsberger et al. 2008). However, many electric fish also possess well developed eyes (Kreysing et al. 2012; Takiyama et al. 2015) that can be used for orientation and even for object recognition (Schumacher et al. 2016). These examples just give us a glance at the importance of multimodal processing in controlling behavior. Due to its behavioral relevance, multimodal processing should be increasingly considered to investigate neural mechanisms of behavior. Fortunately, between 2 and 6% (mean = 4%) of the ICN contributions over the last 13 years followed a multimodal approach (Fig. 1).

## ‘Animal models’ in neuroethology

According to Krogh’s principle (Krogh 1929), each ‘problem’ (i.e., behavior) can be investigated with ‘a few such animals on which it can be most conveniently studied’. Many neuroethologists often advise their students to find the ‘champion’, i.e., the species that is best adapted to the behavior of interest. These species often reveal sensory adaptations that may facilitate finding neural correlates of the behavior. A recent study in birds shows that following Krogh’s principle indeed maximizes the chance of finding sensory adaptations (Payne et al. 2021). The hippocampus, a brain region most extensively studied in rats houses place cells that are important for spatial coding (Moser et al. 2017). When a rat traverses an environment, different subsets of place cells are active in a location-dependent manner. Hence, a population of place cells map the rat’s local environment and are therefore thought to represent a neural correlate for spatial memory. To find the evolutionary origin of place cells, scientists look for place cells in diverse species including fish (Vinepinsky et al. 2017, 2020) and more recently birds (Ben-Yishay et al. 2021; Payne et al. 2021; Agarwal et al. 2023). While attempts to find place cells in quails were unsuccessful (Ben-Yishay et al. 2021), they were eventually discovered in tufted titmice (Payne et al. 2021). As a food-hoarding bird, the tufted titmouse has an extraordinary spatial memory. Importantly, Payne et al. 2021 further demonstrated that chances of finding place cells were reduced in a non-hoarding bird species like the zebra finch. This example not only gives us crucial insights into the evolutionary origin of place coding (Vinepinsky and Segev 2023), but also that chances of finding neural correlates of a behavior are maximized when focusing on the ‘champion’ species that naturally demonstrates the behavior of interest. Altogether, the selection of the model species primarily depends on the behavior of interest and less on the techniques that are available for the species.

**Fig. 2:**
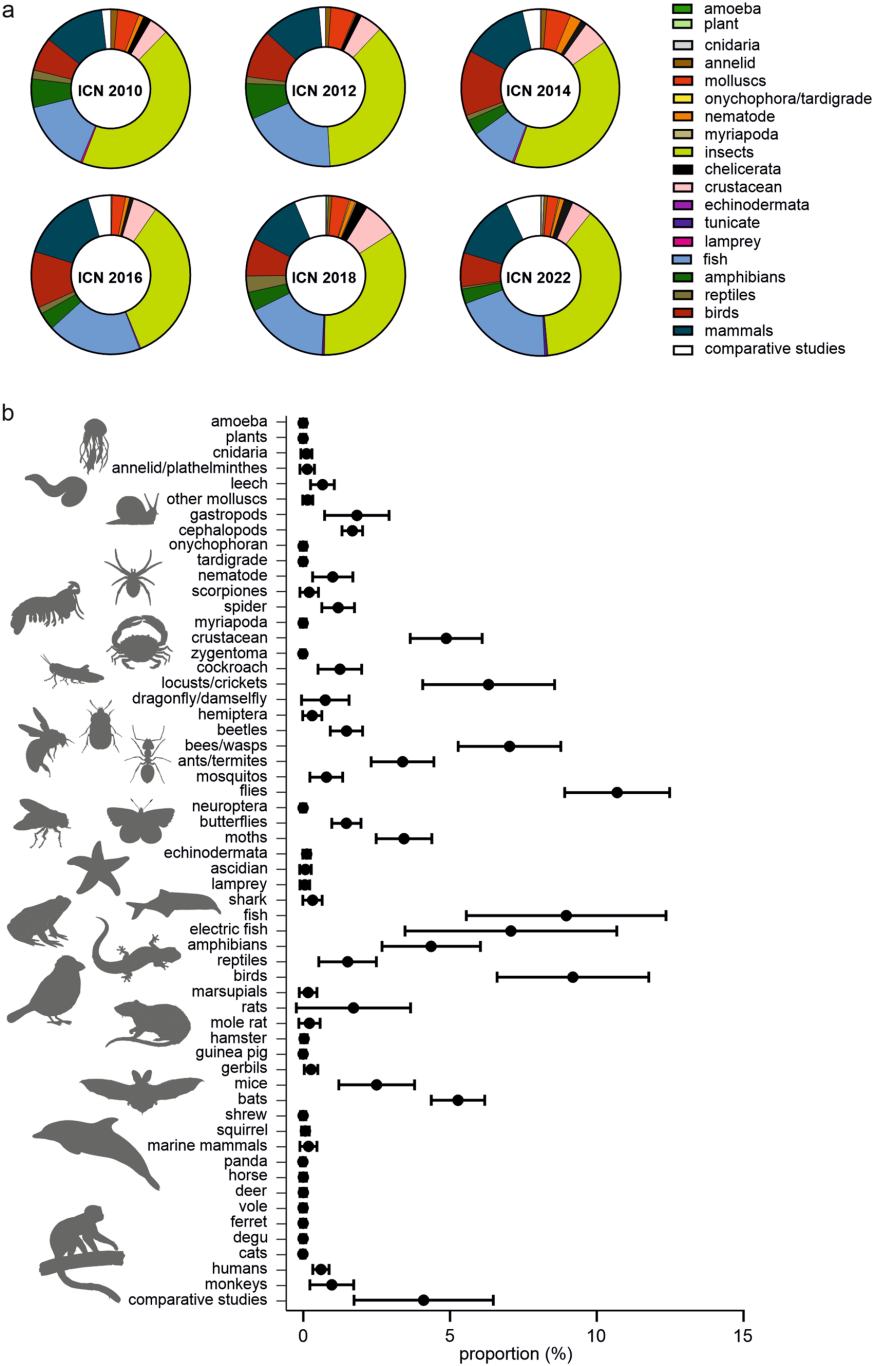
**a** Proportion of model organisms represented at the last six ICNs. Values are listed in Table S2. **b** Detailed distribution of model organisms. Dot and whiskers represent mean and standard deviation, respectively

To determine whether neuroethologists still follow Krogh’s principle, I categorized the ICN abstracts based on the investigated model organisms. Based on Krogh’s principle, the term ‘model organism’ defines the species that is suited to answer a specific neuroethological question. As not every abstract highlighted the investigated species, I used the term ‘taxa’ for categorization. The diversity of taxa represented at the ICN can be appreciated in Fig. 2 which indicates that Krogh’s principle is still followed by many neuroethologists. However, it also becomes clear that some taxa are more represented than others. With about 37% of all contributions, insects are by far the most represented animal group at the ISN (Fig. 2a). More precisely, flies (≈ 10%) represent the biggest group. This bias likely reflects the genetic tools that are available for *Drosophila* and allow scientists to dissect the neural circuits of behavior. Bees and wasps represent the second largest group of insect contributions (≈ 7%). Here, research often focuses on spatial orientation and learning (Menzel 1999, 2023; Menzel et al. 2006). Additionally, the eusocial lifestyle of bees with their age-related polyethism represents another central topic of bee research (Groh and Rössler 2020). The third most represented insect group were locusts and crickets (≈ 6%). Crickets are often used to address questions on acoustic communication (Huber et al. 1984; Pires and Hoy 1992; Ronacher 2019; Römer 2021), while locusts are often investigated in the context of orientation behavior (Beetz et al. 2016c; Beck et al. 2023; Homberg et al. 2023) or locomotion (Dürr et al. 2018; Dürr and Mesanovic 2023). Because of their relatively large size, descending neurons are highly accessible with electrodes enabling an investigation of flight control and looming induced escape behavior in locusts (Rowell 1989; Santer et al. 2006; Duch and Büschges 2022). The dominance of insect research at the ICN is unsurprising because insects represent one of the most diverse and successful animal groups on our planet. Additionally, housing costs are relatively low compared to the animal care of vertebrates. In contrast to these advantages, experiments on insects are technically demanding because of the small body size. Devices for monitoring neural activity are too large to be carried by freely flying insects.

While the proportion of insect research was relatively stable over the last 13 years (33–42%), research on molluscs, particularly on gastropods, progressively decreased from 3.13% in 2012 to 0.46% in 2022. In contrast, the proportion of research conducted in cephalopods was stable between 1.3 and 2.3% (≈ 2%). From my perspective, cephalopods are one of the most fascinating creatures on our planet. Their brain organization is evolutionarily distinct from the rest of the animal kingdom, including their close relatives, and the selection pressure for developing such highly complex nervous systems is still under debate (Albertin and Katz 2023). The octopus brain comprises approximately 500 million neurons, six times more neurons than a mouse brain (Albertin and Katz 2023). About two thirds of the cephalopod brain is devoted to the optic lobes, underlining the dominance of visual processing (Pungor et al. 2023). Cephalopods use vision for diverse behaviors such as navigation, hunting, communication and camouflage (Pungor et al. 2023). For communication and camouflage, chromatophores distributed across the skin, enable cephalopods to dynamically change their visual appearance within a few seconds (Montague 2023; Pungor et al. 2023). To fully merge with their surroundings, cephalopods visually scan the texture of the surroundings to recreate the three-dimensional texture with their skin papillae (Montague 2023). When considering their camouflage skills, it appears paradoxical that cephalopods are actually color-blind (Marshall and Messenger 1996). It therefore remains enigmatic how they accomplish such remarkable camouflage and even fool animals that have high spatial resolution such as humans. The chemotactile sense of cephalopods is also the focus of ongoing research. The cephalopod’s eight independently moving arms are equipped with chemotactile receptors that allow the animal to chemically probe its immediate surroundings (Allard et al. 2023). In addition to their sensory systems, cephalopods' intelligence and behavioral flexibility to solve problems never stops fascinating scientists (Jozet-Alves et al. 2023). The rapid progress in neurogenomics lays the foundation to answer questions on brain (Albertin and Katz 2023) and eye evolution (Nilsson et al. 2023). More recently, the CRISPR-cas system has been used to breed transparent cephalopods (Ahuja et al. 2023), perfect for neuroimaging (Pungor et al. 2023). Additional progress has been made in recording neural activity in cephalopods by using electrodes (Gutnick et al. 2023). The recent technological progress makes cephalopod research a highly promising field in neuroethology.

Among vertebrates, fish are the most frequently investigated group in the ISN (≈ 16%; Fig. 2, Table S2). Fish show a rich repertoire of social behaviors, (Akinrinade et al. 2023) including collective behavior (Jolles et al. 2017) and agonism (Silva et al. 2013; Wayne et al. 2023). In addition, they show fascinating sensory adaptations like electrosensation (Carr 1990; von der Emde 2006) and acoustic communication (Crawford et al. 1986; Hyacinthe et al. 2019; Dunlap et al. 2021). Fish represent the biggest vertebrate group in terms of species number and occupy diverse ecological niches. Genomics and transcriptomics are useful in characterizing molecular mechanisms of social behavior (Renn et al. 2008; Eastman et al. 2020) and the ability to genetically modify zebrafish gives researchers unique opportunities to dissect neural circuits of behavior.

Another ecologically diverse group of vertebrates are the birds. With 9% of ICN contributions, birds are the third most represented vertebrate group (Fig. 2, Table S2). One of the most notable behaviors associated with birds is their vocal behavior, which has been extensively studied in zebra finch (Vicario et al. 2002; Ma et al. 2020; Yu et al. 2020). However, birds are also well known for their migration behavior, another central topic of neuroethology (Chernetsov 2017; Wiltschko 2017). Corvids are often studied because of their cognitive capacities (Veit and Nieder 2013; Breen et al. 2016; Balakhonov and Rose 2017; Rutz et al. 2018), such as their ability to count items (Kirschhock and Nieder 2023), while barn owls are often studied in the context of localizing prey-generated sounds (Carr and Peña 2016; Wagner 2019). Altogether, it seems to be that a ‘champion’ for almost each behavior can be found among birds and their phylogenetic position makes them perfect to answer important questions on the evolution of behavior.

According to the species number, reptiles are as diverse as birds. For each group more than 10, 000 species have been characterized (Uetz et al. 2021). Despite this similarity, reptiles were relatively poorly represented at the last ICN. Less than 2% of ICN contributions from 2010 to 2022 were about reptiles and at the latest ICN in 2022 the proportion even dropped to 0.5% (Fig. 2). This trend is unexpected considering the rich behavioral repertoire and sensory adaptations found in reptiles. Chameleons, for example, can walk along vertical substrates upside-down, independently move their eyes, are highly successful predators, and are known for their camouflage (Ketter-Katz et al. 2020). Sea turtles are famous for their remarkable navigation skills that enable them to reach their home beach years after hatching (Lohmann et al. 2022). Snakes exhibit interesting sensory adaptations for prey detection. Infrared receptors sense the heat radiating from potential prey and allow prey detection in complete darkness (Kaldenbach et al. 2016; Bothe et al. 2019). Despite these fascinating behaviors, scientists often hesitate to work with reptiles. One possible reason could be the reptiles’ low reproduction rate which makes breeding extremely time consuming and expensive. The reptiles’ low metabolic rate further complicates working with reptiles because conditioning experiments with food rewards are quite time consuming. Instead of being frustrated by such pitfalls, some scientists seek to take advantage of them. Because of their low metabolic rate, reptiles are physiologically resistant to extreme hypoxia (Laurent et al. 2016). Some turtles survive periods of hibernation longer than 4 months at 3 °C (Laurent et al. 2016). This inspires research that seeks to understand how nervous tissues tolerate hypoxia (Laurent et al. 2016). In addition, the ‘laziness’ of reptiles is perfect to monitor physiological processes associated with sleep and hence represent enormous potential to investigate evolutionary aspects of sleep (Shein-Idelson et al. 2016; Fenk et al. 2023). Research questions on brain evolution can also be answered by transcriptomics in reptiles (Hain et al. 2022).

Another evolutionarily interesting group of animals are amphibians. Similar to reptile studies, the proportion of ICN contributions on amphibians dropped from 7% in 2012 to 3% in 2014 and since then has stayed at relatively low proportions (Fig. 2, Table S2). Working with amphibians has a long tradition in neuroethology. In particular, acoustic communication in frogs represents a core topic in neuroethology (Narins et al. 2023). Nerve regeneration in axolotl (Lust et al. 2022) and poison resistance in newts are also studied (Hanifin and Gilly 2015; Vaelli et al. 2020). Despite the decreasing number of amphibian contributions, there is growing interest in investigating neural mechanisms of cognitive processes (Liu et al. 2019; Khatiwada and Burmeister 2022) or social behaviors such as parental care in poisonous frogs (Fischer 2023; Moss et al. 2023). To this end, scientists not only use behavioral assays but also transcriptomics to find the molecular mechanisms of social behavior (Fischer et al. 2019, 2021). Transcriptomics are also quite useful in shedding light on the evolution of the amphibian brain (Woych et al. 2022). Altogether, amphibians provide many interesting questions for neuroethologists, and thus have great potential to influence the future of neuroethology.

Bats are the mammals that were most represented (≈ 5%) at the last ICNs, with the exception of the congress held in 2016. Bat research represents a good example of how neuroethological topics change over the years. Traditionally, bats were investigated to understand how they negotiate obstacles in darkness without using their eyes (Simmons et al. 1978; Neuweiler 2003; Beetz and Hechavarría 2022). This remarkable feat, which is based on a highly specialized auditory sense, has fascinated scientists for decades. With the increasing knowledge of acoustic processing, some bat researchers have shifted their focus towards acoustic communication (Salles et al. 2019). As highly social animals, bats live in colonies and exhibit a huge repertoire of different communication signals. It is not only interesting how communication signals are processed in the bat brain but also how they are segregated from echolocation signals (López-Jury et al. 2021). By taking a closer look at the ontogeny of vocalization signals, scientists have found that pups hand-reared in the absence of conspecific vocalizations, but in the presence of an experimentally controlled bat call, fine-tuned their social call to the play-backed call. This phenomenon was not observed in pups that were not acoustically stimulated (Esser 1994). These early findings triggered research on vocal learning in bats which is meanwhile a central topic in neuroethology (Vernes and Wilkinson 2020).

Nachum Ulanovsky, who has a keen interest in understanding the spatial representation in the bat hippocampus, has taken a different research direction. When he was postdoctoral researcher in Cynthia Moss’ laboratory, research on spatial representation in the hippocampus was predominantly conducted in rodents that oriented in laboratory mazes. Nachum Ulanovsky, however, followed a neuroethological approach and aimed to study the spatial code in bats that orient under more natural conditions. By recording from the hippocampus of crawling bats (Ulanovsky and Moss 2007), his laboratory meanwhile investigates spatial coding in freely flying bats that navigate hundreds of meters in a tunnel (Eliav et al. 2021). What else could we learn from bats in the future? When I worked with bats during my PhD, I was astonished by the longevity of bats. Although they exhibit a high metabolic rate comparable to mice, it is not rare to find 10 or even 15-year old bats (Brunet-Rossinni and Austad 2004; Wilkinson and Adams 2019). The physiological processes that may explain why bats became the Methuselah of small mammals still await to be discovered and could be applied to age-related neuro-degenerative diseases, such as dementia.

## Importance of comparative studies

In contrast to the approach of systems neuroscience, neuroethology seeks to investigate behavior in diverse species (Bleckmann 2023). If physiological processes are conserved across the evolutionary scale, we can make general statements. At the same time, physiological adaptations that are distinct in a few species help us not only to understand how the nervous system works, but also how subtle modifications in the neural circuit may affect behavior. To this end, comparative approaches are essential to understand how nervous systems operate and how they evolved. This can be appreciated by recent findings in the mammalian hippocampus. The activity of place cells oscillates in the theta range (Buzsáki 2002). These brain oscillations had been suggested to have different functions such as neural communication (Colgin et al. 2009), memory (Lisman John and Jensen 2013), and navigation (Burgess and O'Keefe 2011). Unexpectedly, a theta rhythmicity could not be detected in monkeys (Hori et al. 2011), nor in flying bats (Yartsev and Ulanovsky 2013), suggesting that theta oscillations are not essential for place coding. This example demonstrates that results can only be circumscribed as ‘fundamental’ when the mechanisms are conserved across diverse species. Fortunately, neuroethologists show a keen interest in following a comparative approach. This is reflected by an increase in ICN contributions that work with multiple different species, from 1% at the ICN in 2012 to 7% at the ICN in 2022 (Fig. 2, Table S2).

## Research topics

To get an overview of the current research directions of the field, I categorized the ICN abstracts into different research topics. Because of the diversity of topics, it was challenging to determine a limited number of research topics. I decided against using topic categorizations as defined by the ICNs because each congress had a slightly different categorization, making a direct comparison difficult. To acknowledge the diversity of research topics, I named 55 research topic categories. A brief explanation for each category is given in the glossary. I assigned research topics to each abstract based on its content. I often assigned multiple topics to a single contribution. For example, frog studies often focus on acoustic communication in the context of mating, and some studies additionally investigate hormones. In that case, I assigned three topics, i.e., ‘communication’, ‘courtship behavior’, and ‘hormones’. To also highlight technological advances, I included some methodological categories, such as the use of virtual realities or tracking techniques. Together, with the quantification of contributions to the Journal of Comparative Physiology-A/Zeitschrift für vergleichende Physiologie (Wagner et al. 2024), we may get a representative view of the recent past, as well as the present status, of neuroethology.

## Spatial orientation, memory, and communication are the top 3 research topics

**Fig. 3:**
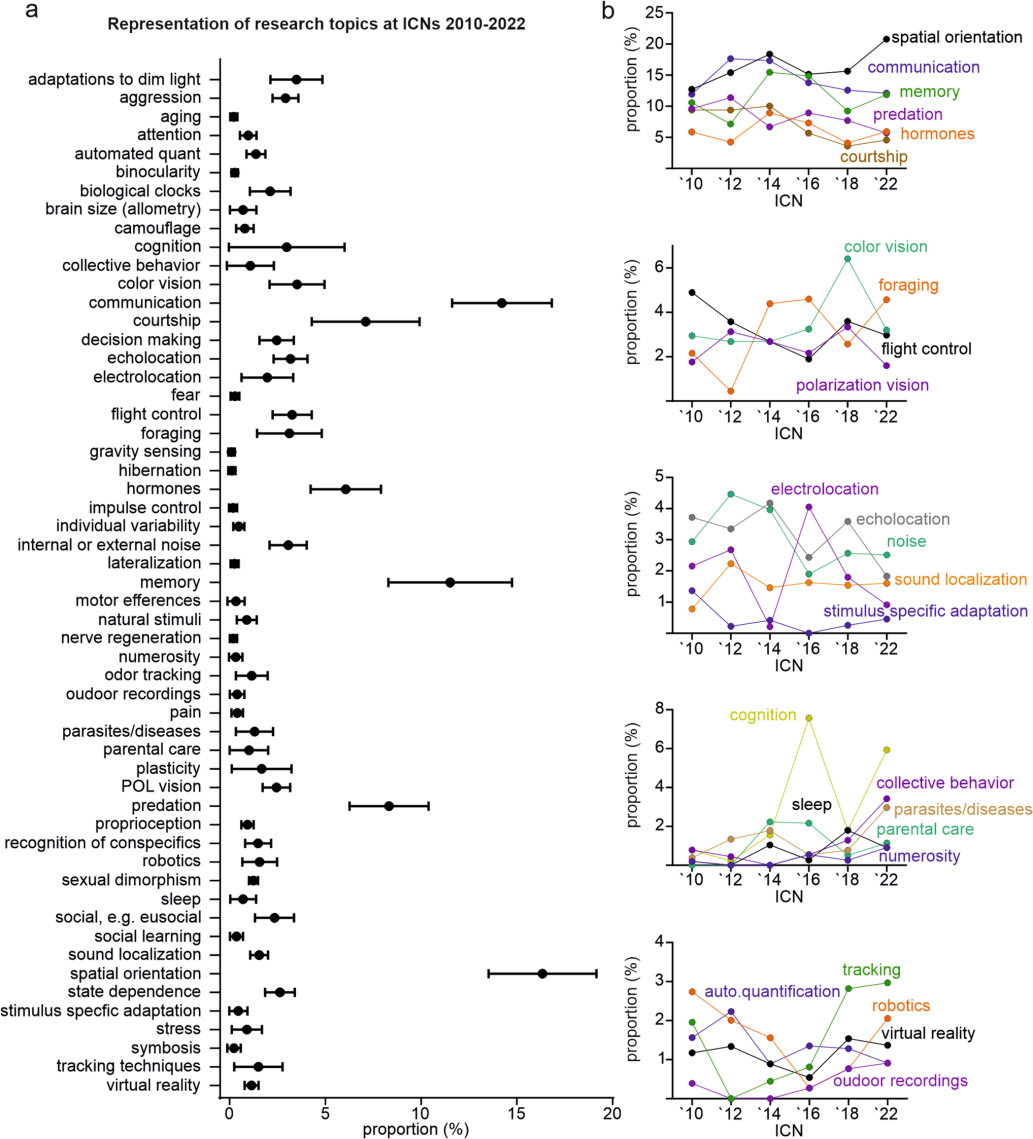
Representation of research topics at the last six ICNs.** a** Distribution of 55 research topics. For topic descriptions see the glossary. Dot and whiskers represent mean and standard deviation, respectively. **b** Proportional change of selected research topics over the last twelve years. *POL* polarization, *quant* quantification

A substantial fraction of ISN delegates work on spatial orientation (≈ 16%; Fig. 3a, Table S3). Over the last decade, this topic has attracted many neuroethologists (Fig. 3b). While 13% of ICN contributions in 2010 were about spatial orientation, more than one fifth of the abstracts (21%) covered this topic at the last ICN in 2022. A keen interest in this topic is not surprising when considering the evolutionary conservation of spatial orientation. In essence, every species that actively moves must need some sense for orientation. Orientation behavior is relevant across different spatial scales from just a few centimeters as it is the case for thermotaxis in *C. elegan*s (Mori and Ohshima 1995), to thousands of kilometers as seen in many migratory species (Mouritsen 2018). Depending on the spatial scale, different sensory modalities are used for spatial orientation. In most cases, however, spatial orientation is based on multimodal processing. This complicates research on spatial orientation, but further drives the fascination for this topic. While neural activity can be monitored in diverse species and under well-controlled laboratory conditions, it remains a mystery as to how the brain operates when an animal orients over several kilometers in its natural habitat. The development of ultralightweight recording devices undoubtedly advances the field of navigation and will allow scientists to unravel the secrets of animal navigation (Gaidica and Dantzer 2022; Givon et al. 2022; Ide and Takahashi 2022; Menz et al. 2022).

For efficient orientation in a familiar habitat, many species form a spatial memory which can be represented by vectors, as it is the case in insects (Webb 2019), or as a spatial layout of the environment, a so called ‘cognitive map’ (Whittington et al. 2022; Farzanfar et al. 2023). Understanding neural processes of spatial memory is a central topic in neuroethology (Menzel et al. 2006; Geva-Sagiv et al. 2015; Ardin et al. 2016; Stone et al. 2017; Webb 2019). However, spatial memory reflects only a small portion of memory research. Memory is also essential for communication, another ‘hot topic’ of neuroethology (Fig. 3a, b). On average 14% of ICN contributions from 2010 to 2022 were about communication with many of them covering vocal learning (Knörnschild 2014; Jarvis 2019; Vernes et al. 2021). Communication is crucial for our daily life and understanding its neural underpinnings, especially in the context of vocal learning is essential. Comparative research beautifully demonstrates the importance of auditory feedback for vocal learning (Brainard and Doupe 2000; Smotherman et al. 2003; Tschida and Mooney 2012) such as in the case of the sensitive phase in infants in order to learn how to vocalize.

Communication is not only based on acoustic signals. Weakly electric fish, for example, use self-generated electric fields for communication (Zupanc et al. 2006; Jones et al. 2021; Caputi 2023). Depending on the species, these electric signals are sine waves or pulses that are reproducible in the laboratory. This allows scientists to ‘communicate’ with electric fish and perturb the fishes’ electric sense, an elegant approach to investigate the active electric sense. Electric fish also use their electric sense for short-range orientation, referred to as electrolocation (Caputi and Budelli 2006; von der Emde 2006). Objects surrounding the fish distort the electric field which can be sensed by electroreceptors distributed across the fish’s skin. The larger and closer the object is, the more electroreceptors sense the distortion (Haggard and Chacron 2023). In addition to localizing close-by-objects, the electric sense allows the fish to discriminate between living and non-living objects. Depending on the conductivity of objects, electric fields get differently distorted, which allows a rapid categorization between a stone or a potential prey. While 4% of the ICN 2016 contributions were about electrolocation, the proportion decreased to less than 1% at the ICN in 2022 (Fig. 3b). This reflects the current situation of electric fish research which seems to focus more on aspects of electric communication and antagonistic behavior and its hormonal control (Perrone and Silva 2018; Metzen 2019; Dunlap et al. 2021). The ability to monitor brain activity in freely swimming fish (Takahashi et al. 2021; Cohen et al. 2023) has a great potential for investigating electrolocation under more natural conditions in the future.

## Technical approaches

Many neuroethologists work on species that cannot be genetically modified. To overcome these technological constraints, there is strong effort in developing genome editing tools in different species (Dierick et al. 2021). The CRISPR-Cas system represents a broadly used gene-editing tool in neuroethology (Dierick et al. 2021; Alward and Juntti 2023). Since the discovery of its potential, scientists have edited genes in diverse species including cephalopods (Ahuja et al. 2023), butterflies (Markert et al. 2016; Livraghi et al. 2018), beetles (Gilles et al. 2015), fish (Barske et al. 2020; Bedbrook et al. 2023), axolotl (Fei et al. 2018), lamprey (Square et al. 2015), lizards (Rasys et al. 2019), and opossums (Kiyonari et al. 2021). Genome editing is not only used to knockout genes, but it also helps to monitor the activity of anatomically traceable neurons. With the PiggyBac transposon system (Schulte et al. 2014), scientists recently generated a pan-neuronal genetic driver in the honeybee (Carcaud et al. 2023). By expressing a calcium indicator under the control of a presynaptic protein (synapsin) promotor, scientists simultaneously imaged the neural activity in multiple brain regions upon olfactory stimulation (Carcaud et al. 2023). Although these recordings were conducted in restrained bees, this technique could potentially be combined with a virtual reality setup that allows the insect to interact with a visual scene under closed-loop settings. The use of virtual realities has a long tradition in insect science (Reichardt 1973; Heisenberg and Wolf 1979). One of the early studies that used a virtual reality setup to investigate visual pattern learning in *Drosophila* had been published in Journal of Comparative Physiology-A (Wolf and Heisenberg 1991). At that time, a fly was attached to a torque meter at the center of a cylinder whose inner wall was equipped with visual patterns. The torque meter measured the fly’s intended rotatory movements which correspondingly adjusts the angular position of the cylinder. Over the years, these settings substantially changed. Instead of mechanically moving a cylinder, a visual pattern displayed on a circular LED screen can be rotated, depending on the fly’s orientation behavior (Reiser and Dickinson 2008). In addition, the torque meter had been replaced by a video camera that monitors the wing beat amplitude from both wings. Based on the wing beat amplitude difference between left and right wing, the fly’s intended steering direction can be measured, and the LED screen gets accordingly updated. While these virtual realities are limited to simulate the insect’s rotatory movements, more recently developed setups additionally simulate translatory (i.e., forward and backward) movements of the insect (Haberkern et al. 2019; Kaushik et al. 2020). One of the most sophisticated virtual realities used in insects, but also in vertebrates, are settings that create a closed loop between a visual scene and a freely moving animal (Stowers et al. 2017). However, monitoring brain activity from an insect that is moving freely currently works only with extracellular recording techniques (Martin et al. 2015; Jin et al. 2020; Wosnitza et al. 2022). In contrast, in freely moving vertebrates, brain activity can be monitored with ultralight microscopes attached to the scalp (Düring et al. 2020; Liberti et al. 2022; Zong et al. 2022; Hasani et al. 2023). Despite these technological advances, the proportions of ICN contributions focusing on virtual realities were stable over the last decade (1%, Fig. 3b), a situation that may change when neuroethologists combine virtual realities with neural recordings in genetically non-tractable species.

Instead of conducting research under constrained laboratory conditions, we should always evaluate the possibility of moving from the lab into the field (Hoffmann et al. 2023). Field experiments, however, come with challenges. For example, stimuli in the field are much more diverse than the ones tested in the laboratory. In the field, stimulus scenarios are usually unique and cannot easily be replicated. This makes it impossible to compare neural data collected in the field with conventional methods that had been conceptualized under the premise that the data were collected in response to many identical trials (Miller et al. 2022). However, this analytical concept is miles away from the natural situation when a brain must reliably work at any moment in time and initiate the behavior that is most appropriate for the current situation. Not only the quantity of stimulus replicates is unpredictable in the field, but also the quality, i.e., the stimulus salience. Since the beginning of electrophysiology, scientists have presented salient stimuli in absence of any potentially interfering stimuli. An auditory scientist for example performs experiments in a sound-proof room. While this approach undoubtedly gives insights into the principles of neural coding, under these conditions it is challenging to test how the neurons process naturalistic stimuli that are embedded in a sensory stream. Fortunately, neuroethologists were aware of this problem early on and this may also be the reason why a substantial fraction of 3 ± 0.9% of ICN contributions investigated how potentially interfering stimuli may mask the neural response to the behaviorally relevant stimulus (topic: ‘internal or external noise’ Fig. 3, Table S3). Understanding how the nervous system processes more naturalistic stimulus scenarios is essential to interpret brain activity recorded in the field. For example, the extensive literature on the neural processing of bird songs (Keller and Hahnloser 2009; Fortune et al. 2011; Theunissen and Elie 2014; Moore and Woolley 2019; Das and Goldberg 2022) paved the way to monitor brain activity from vocalizing birds in the field (Hoffmann et al. 2019; Coleman et al. 2021). It is therefore encouraging to see an increasing trend testing how naturalistic stimuli are processed in the brain (from 0.2% in 2010 to almost 1.4% in 2022, Fig. 3, Table S3) and performing recordings outdoors (from 0.3% in 2016 to almost 1% in 2022).

While conducting field experiments, it is not only the sensory stimuli that vary but also the animal’s internal state. Internal states, such as hunger, arousal, and hormonal levels, not only substantially affect behavior but also neural activity (Abbott 2020; Kanwal et al. 2021). To decode the recorded brain activity, scientists must conduct experiments under diverse internal states. This could be achieved with long-term recordings. The development of mesh-structured electrodes allows scientists to perform life-long neural recordings (Dhawale et al. 2017; Luo et al. 2020; Steinmetz et al. 2021; Guan et al. 2023; Zhao et al. 2023). Measuring brain activity in the field is just one side of the coin. The neural data must also be related to the animal’s behavior. To this end, the behavior must be tracked in sufficient detail, which is not trivial when considering small, highly mobile animals. Fortunately, camera systems have improved over the years and allow scientists to precisely track animals (Vo-Doan and Straw 2020; Fabian et al. 2024). In addition, video analyses are also improving. While reflective markers had to be mounted on the animals in the past, state-of-the-art tracking software, such as DeepLabCut (Nath et al. 2019; Mathis et al. 2020), Ctrax (Branson et al. 2009) or DeepPoseKit (Graving et al. 2019) enable marker-free pose tracking in diverse species. Camera-based tracking approaches may not be ideal to track animals in highly cluttered environments. Under these conditions, radar-based methods may be favored. Radar tracking is widely used and transmitters/transponders that must be mounted on the animal are meanwhile so tiny so that they barely interfere with the animal’s behavior. Radar tracking however bears limitations. For example, it reveals only a two-dimensional coordinate, and it does not reach the spatio-temporal accuracy of video tracking. Altogether, each tracking method must be carefully evaluated before moving from the lab into the field. Fortunately, technological progress is rapid (Daniel Kissling et al. 2014; Nourizonoz et al. 2020; Vo-Doan and Straw 2020; Lioy et al. 2021; Shearwood et al. 2021; Walter et al. 2021; Gaidica and Dantzer 2022; Raab et al. 2022; Nagy et al. 2023), which is reflected by ICN contributions working on tracking techniques. These contributions have increased since the ICN in 2014 from 0.45% to almost 3% (Fig. 3b).

## Glossary

**Adaptations to dim light**: This topic deals with research focusing on sensory adaptations to dim light. It includes research on nocturnal vision and additional sensory adaptations related to dim light conditions.

**Aggression**: Resources, such as space, food, or mates, are limited. This limitation often results in aggressive behavior which is central to many neuroethological projects.

**Aging**: This topic includes research related to the molecular mechanisms of aging.

**Attention**: This broad term refers to ICN abstracts that explicitly stated ‘attention’.

**Automated quantifications**: This topic refers to any tool that enables an automated quantification. Many behavioral studies on *Drosophila* monitored the behavior of single flies with the goal to fully describe the behavioral repertoire.

**Binocularity**: This topic investigates the neural computations of stereo vision.

**Biological clocks**: This topic refers to research that focuses on biological rhythms.

**Brain size**: This topic includes research that relates brain sizes to behavioral or sensory adaptations.

**Camouflage**: This topic refers to research on the neural mechanisms of camouflage.

**Cognition**: Under this broad term, I assigned only abstracts that explicitly stated ‘cognition’.

**Collective behavior:** This research topic refers to projects focusing on collective behavior such as swarm intelligence.

**Color vision**: This topic includes research that focuses on the discrimination of wavelengths.

**Communication**: This topic includes any kind of communication between at least two conspecifics. While most research focuses on acoustic communication, other sensory modalities used for communication are also included in this topic.

**Courtship behavior**: This topic includes research that deals with mating or courtship behavior. This also includes research on acoustic communication in frogs or crickets whose ultimate goal is to localize potential mates.

**Decision making**: This broad topic only contains abstracts that explicitly stated ‘decision’.

**Echolocation**: This topic refers to research on the biosonar of marine mammals or bats.

**Electrolocation**: This topic includes research on active and passive electrolocation. In contrast to passive electrolocation in which animals perceive electric fields emitted from surrounding organisms, active electrolocation refers to the ability to produce electric discharges for orientation purposes.

**Fear**: This topic refers to research on the effects of fear on the nervous or hormonal systems or on behavior.

**Flight control**: This topic includes research that focuses on the biomechanics of flight, especially on how visual feedback is important for flight control.

**Foraging**: Finding food is essential for survival. This category refers to the research focusing on foraging.

**Gravity sensing**: This topic refers to research focusing on how animals sense gravity.

**Hibernation**: This topic refers to research focusing on the physiological processes involved in hibernation.

**Hormones**: This topic includes research focusing on the hormonal control of neural processing and behavior.

**Impulse control**: Most research represented at the ICN on impulse control investigated impulse control in domestic chicks (Amita et al. 2010).

**Individual variability**: This topic refers to research that aims to understand the inter-individual variability of behavior.

**Internal/external noise**: Behaviorally relevant stimuli are usually embedded in non-relevant stimuli. This topic focuses on research on signal processing in the presence of potentially interfering stimuli. In addition, neurons often show an inter-trial variability, i.e., the presentation of a stimulus does not always evoke the same neural response. Research focusing on how neurons convey information despite the inter-trial variability are categorized into ‘internal noise’.

**Lateralization**: This topic refers to research that focuses on right-left brain asymmetries or lateralization of a behavior.

**Memory**: This topic represents a broad spectrum of research such as vocal learning, visual memory in the context of spatial orientation, but also molecular mechanisms of learning and memory.

**Motor efferences**: Any voluntary movement creates an internal copy of the motor pattern (efference copy) that allows a prediction of the sensory input. This prediction may for example be used for flight stabilization.

**Natural stimuli**: This topic represents research that uses naturalistic stimuli, e.g., communication calls.

**Nerve regeneration**: Some species show remarkable abilities to regenerate neuropathic injuries. This topic refers to research that focuses on the molecular mechanisms of nerve regeneration.

**Numerosity**: This topic refers to research that focuses on numerical discrimination, a behavior not restricted to vertebrates [see (Howard et al. 2019; Bengochea et al. 2023)].

**Odor tracking**: Many animals, such as moths locate potential mates by tracking pheromones. This topic also refers to research dealing with host seeking behavior based on olfactory cues.

**Outdoor recordings**: This topic includes projects that use electrophysiology outdoors in the field. In this topic, I also include measurements of electric organ discharges in the natural habitat of electric fish.

**Pain**: This topic refers to research on nociception.

**Parasites/diseases**: This topic includes research on parasites and diseases that affect the nervous system (including behavior).

**Parental care**: This topic refers to research that investigates parental care. 

**Plasticity**: This topic includes research on age-related synaptic plasticity often observed in the context of age polyethism in eusocial insects.

**Polarization vision**: Many species use polarization information for diverse behaviors such as orientation or communication. This topic refers to research that investigates the neural mechanisms of polarization vision.

**Predation**: This topic refers to research on hunting or escape behavior. Many of the projects use looming stimuli to evoke escape behavior and to understand its neural mechanisms.

**Proprioception**: This topic refers to research that focuses on how animals receive sensory feedback from their body, e.g., body postures.

**Recognition of conspecifics**: This topic includes research on recognizing conspecifics or nest mates. Recognition of conspecifics is crucial, for example, for eusocial insects that must discern nest mates from potential intruders.

**Robotics**: This topic is dedicated to research that aims to transfer empirical data into robots to test the behavioral relevance of the neural findings.

**Sexual dimorphism**: This topic refers to research on sex specificity. This includes sexual dimorphism at anatomical, physiological, or behavioral levels.

**Sleep**: This topic includes research on the neuroethology of sleep.

**Social behavior**: This topic includes research that aims to understand the neural mechanisms of eusocial behavior. In addition, many studies on fish investigate a diverse repertoire of social behaviors without giving explicit details in their abstracts. These together with studies on eusocial insects are represented in this topic.

**Social learning**: Many species learn from conspecifics, often referred to as observational learning. Abstracts focusing on observational learning from conspecifics are categorized here.

**Sound localization**: Localizing sound sources is critical for many behaviors such as finding prey in the example of nocturnal birds or bats, or a potential mate as it is the case for frogs or crickets.

**Spatial orientation:** This topic includes research that focuses on goal-directed movements.

**State dependence:** Behavior and neural processing strongly depends on the animal’s state, e.g., whether it is quiescent, moving. In addition, the animal’s internal state (e.g., hunger, alertness), determine the motivation to show a particular behavior. This topic refers to research that focuses on state dependence.

**Stimulus specific adaptation**: Neurons often adapt to repetitive stimuli, i.e., the neural response decays with the number of stimulus presentations. The sensitivity to rarely occurring stimuli however is preserved. Research dealing with this specific adaptation was grouped into ‘stimulus specific adaptation’ (SSA), a field that often focuses on auditory signals.

**Stress**: This topic includes research on the influence of stress on the brain and behavior.

**Symbiosis**: This topic refers to research that investigates symbiosis, i.e., a social interaction from which at least two species profit from.

**Tracking techniques**: This topic refers to tools that aim to track the position or the posture of animals.

**Virtual reality**: This topic refers to research that uses virtual realities to answer neuroethological questions.

## Research highlights

For the remaining part of this perspective, I highlight three research topics that have recently captured my interest and that will hopefully gain more attention in the neuroethology community in the future. Due to space constraints, I focus here on three topics, which does not mean that other neuroethological research topics are less fascinating.

## Neural correlates of social interactions

Whether a male cricket sings to attract a female or a pup calls for her mother in a bat colony, each individual is interacting with conspecifics. Characteristic for such social interactions is that at least two individuals form a sensory-motor loop. For example, acoustic signals emitted by a male cricket are detected by a female which initiates a phonotactic behavior. In other words, the sensory information received by the female elicits a corresponding motor output. Although social behavior implies the engagement of multiple individuals, neural recordings are usually conducted in single individuals. To fully understand the neural mechanisms of social behavior, it is essential to record simultaneously from multiple individuals. Recently, scientists managed to record from a pair of vocally interacting birds (Hoffmann et al. 2019; Coleman et al. 2021). These recordings revealed that auditory feedback from the conspecific is crucial to synchronize the neural activity in premotor brain regions. Importantly, this neuronal synchrony was not observed in anaesthetized birds emphasizing the importance of the sensory loop between two individuals (Fortune et al. 2011). Synchronized brain activity between two interacting individuals has also been demonstrated in communicating bats (Zhang and Yartsev 2019). However, in addition to neural synchrony, there were also rapid fluctuations of activity differences between the two individuals (Zhang et al. 2022). Both examples focused on acoustic communication. Whether neural synchrony can also be observed in communications based on other sensory modalities, for example in electrocommunication or visual communication (such as during the courtship behavior of jumping spiders), remains unanswered. In addition to conducting neural recordings in pairs of individuals, it may also be interesting to see if and how the brains of animals in groups, such as in swarms, are synchronized to each other. The neuroethology of collective behavior represents an exciting research topic, and the combination of virtual realities, computational simulations, and neural recordings may be a promising approach to unravelling the secrets of collective behavior (Couzin 2009; Sridhar et al. 2021).

## Neural mechanisms of sleep

Traditionally, neuroethologists get inspired by observing animals in the field. But what about behavior that is difficult to observe because it is only mentally represented? For example, what happens in the brain when we are sleeping? According to the four criteria of sleep [quiescence, increased arousal threshold, rapid reversibility, and homeostasis (Campbell and Tobler 1984)], sleeping behavior has been described in diverse species (Lakhiani et al. 2023) including cnidarians (Nath et al. 2017; Kanaya et al. 2020), molluscs (Vorster et al. 2014), and insects (Kaiser and Steiner-Kaiser 1983; Hendricks et al. 2000; Shaw et al. 2000). Sleep is often biphasic with alternating periods of quiescence and active sleep. Active periods are described as rapid eye movement (REM) phases (Aserinsky and Kleitman 1953), which humans often experience as story-like dreams (Hobson 2009). While REM phases have also been reported in non-mammals, such as spiders (Rößler et al. 2022) and reptiles (Shein-Idelson et al. 2016), it remains unclear whether these animals dream. Answering this question might help us to understand the function of sleep. One proposed function is that sleep is important for memory consolidation (Smith et al. 1974; Stickgold and Walker 2013). During sleep the activity of our brain oscillates, a neural signature common to many species (Shein-Idelson et al. 2016; Yamazaki et al. 2020). In spite of these physiological parallels, we still cannot assess whether non-human species dream. However, we can test whether sleep promotes memory consolidation in different species. For example, when a sleeping bee gets stimulated with a previously learned odor, her memory improves compared to a honeybee that is not exposed to the odor while sleeping (Zwaka et al. 2015). To characterize the neural mechanisms of memory consolidation associated with sleep, scientists monitored the brain activity of resting rats that had recently explored a novel environment. While recording from the hippocampus, they noticed that the neural activity of a population of place cells was often replayed in a time-compressed manner when the rats were sleeping (Wilson and McNaughton 1994; Frankland and Bontempi 2005). Interestingly, these replays were associated with memory formation (Ji and Wilson 2007; Drieu et al. 2018; Huelin Gorriz et al. 2023). Similar memory-associated replays have been characterized in birds (Shank and Margoliash 2009). In birds, sleep induces spontaneous bursting activity of premotor neurons that reflect neural activity about daytime vocal learning. More importantly, changes in night-time neural activity preceded the onset of practice-associated neural activity in vocal learning in zebra finches. While these findings undoubtedly demonstrate the importance of sleep on memory consolidation, sleep research must be conducted in the field, to fully appreciate its ecological relevance and possibly explain species-specific trade-offs (Rößler et al. 2022). I am convinced that there are methodological techniques available to investigate the neuroethology of sleeping behavior and its function in diverse species, and also in more naturalistic contexts.

## ‘Mind-controlling’ parasites

Parasites and their hosts have coevolved fascinating behaviors. After infecting their hosts, some parasites control their host behavior to promote their own fitness (Libersat et al. 2009; Libersat and Gal 2013; Hughes and Libersat 2019). This can lead to extreme cases where the host loses its control over its body, although the motor systems are not paralyzed; a situation described as zombification. One zombification that has gained a lot of attention is the interaction between the jewel wasp and its host a cockroach (Williams 1942; Haspel et al. 2005; Catania Kenneth 2018). The jewel wasp’s goal is to oviposit an egg on a cockroach, which will serve as a protein-rich source for newly-hatched larvae. To manipulate the behavior of a cockroach, the wasp zombifies the cockroach. To this end, the wasp stings the cockroach’s first thoracic ganglion. This paralyzes the cockroach so that the wasp can direct her sting with remarkable precision into the cockroach’s motor command center (central complex), in the central brain (Haspel et al. 2003). To guide the sting into the central complex, the jewel wasp may receive somatosensory feedback from the sting (Gal et al. 2014). After injecting the venom into the central brain, the paralysis wears off, and occasionally transforms the cockroach into a submissive zombie. In this state, the cockroach does not attempt to escape, despite not being paralyzed (Emanuel and Libersat 2017). Then, the wasp drags the cockroach into a nearby burrow where she stings the cockroach another four times (Catania Kenneth 2020). This last sequence of stings is directed at the second thoracic ganglion and induces an extension of the cockroach’s femur to expose the desired oviposition site for the jewel wasp (Catania Kenneth 2020). To understand the neural mechanisms of this behavior, scientists require sophisticated knowledge on the insect’s motor system, the jewel wasp’s ability to localize a host, and the injection sites. Finally, how the venom physiologically affects the cockroaches’ nervous system is another central question (Emanuel and Libersat 2017).

Because neural activity can be monitored in freely moving cockroaches (Martin et al. 2015), it is technically feasible to dissect the effects that the venom has on the nervous system. Ultimately, this will help us to understand how behavior can be pharmacologically manipulated. It is noteworthy that this is just one example of zombification. There are many more examples where parasites hijack the behavior of their hosts at the expense of the host’s fitness. For example, crickets zombified by nematodes commit suicide by leaping into bodies of water (Thomas et al. 2002). Fungi can also control hosts’ behavior (Hughes et al. 2011; Hughes and Libersat 2019). The fungus *Entomophthora muscae*, for example, infects flies including *Drosophila melanogaster*, and induces a behavior called ‘summit disease’ which is characterized by the host climbing up a substrate (MacLeod et al. 1976; Elya et al. 2023), possibly to be more conspicuous to predators (Martín-Vega et al. 2018). The involvement of *Drosophila* in this parasite-host interaction allows scientists to use genetic tools to dissect the neural circuit responsible for zombification (Elya et al. 2023). With this diversity of parasite-host interactions, it is only a matter of time before more neuroethologists join this interesting field of research to investigate the neuroethology of zombification. This trend could already be seen at the ICN in 2022 where host-parasite interactions had been investigated in almost 3% of the conference contributions, compared to 0.4% at the ICN in 2010 (Fig. 3, Table S3).

## Concluding remarks

Hopefully, this perspective not only gives an overview of the recent scientific progress in neuroethology but also insights into the field’s philosophy. Neuroethology stands for comparative research where scientists working on diverse species often find their common ground in the behaviors that drives their fascination. For example, I currently study how the insect brain represents space and I get inspired from both invertebrate and vertebrate research. Personally, I do not identify my research with the species I am working with, but rather the behavior that triggers my enthusiasm as a scientist. It is therefore essential for me to discuss research with scientists working with diverse animal models and sensory systems. I strongly encourage comparative approaches to assess the fundamentality of the findings. At the same time, investigating adaptations in ‘champion’ species is crucial to find neural correlates of behavior and how slight modifications of a neural circuit affect behavior. Since its launch 100 years ago, the Journal of Comparative Physiology-A captures the enormous diversity of neuroethology. The future of neuroethology strongly depends on the scientific exchange across disciplines—which is granted by this journal—and the development of techniques. Diverse techniques are necessary to investigate animal behavior at different levels, from hormones and neural activity to a detailed analysis of the behavior. Current tools enable precise monitoring of neural activity and behavior allowing scientists to pursue the neuroethological dreams of conducting experiments in the field where it can be best related to natural behavior. I am convinced that more neuroethologists will fulfill this dream in the coming years, and that the Journal of Comparative Physiology-A will accompany their scientific journey.

### Supplementary Information

Below is the link to the electronic supplementary material.

Supplementary file1 (DOCX 28 KB)
