# Supplementary material for: A perspective on neuroethology: what the past teaches us about the future of neuroethology
Source: J Comp Physiol A Neuroethol Sens Neural Behav Physiol. 2024 Feb 27;210(2):325–46. doi: 10.1007/s00359-024-01695-5 (PMC10995053; doi:10.1007/s00359-024-01695-5)
Supplement: Supplementary file 1 — Supplementary file1 (DOCX 28 KB) [file 359_2024_1695_MOESM1_ESM.docx]

**Table 1:** Proportions (in %) of sensory and motor systems represented at the last ICNs.

| **Sensory modalities and motor system** | **International congress for neuroethology** | | | | | |  |
| --- | --- | --- | --- | --- | --- | --- | --- |
|  | **2010** | **2012** | **2014** | **2016** | **2018** | **2022** | **mean** |
| vision | 25.83 | 27.46 | 23.38 | 25.41 | 38.97 | 29.22 | 28.38 |
| motor system | 12.33 | 14.29 | 19.00 | 11.35 | 18.46 | 14.61 | 15.01 |
| audition | 18.98 | 20.54 | 14.82 | 11.08 | 12.56 | 11.42 | 14.90 |
| chemosensation | 11.94 | 6.92 | 10.86 | 11.08 | 6.15 | 9.82 | 9.46 |
| somatosensation | 8.81 | 6.92 | 3.34 | 2.70 | 4.36 | 3.20 | 4.89 |
| aquatic electrosensation | 8.02 | 9.15 | 2.51 | 11.08 | 3.33 | 5.48 | 6.60 |
| terrestrial electrosensation | 0.00 | 0.22 | 0.21 | 0.27 | 0.51 | 0.91 | 0.35 |
| magnetosensation | 0.39 | 0.89 | 0.63 | 1.62 | 1.03 | 2.28 | 1.14 |
| thermosensation | 0.00 | 0.45 | 1.25 | 0.27 | 0.00 | 0.68 | 0.44 |
| hygrosensation | 0.00 | 0.00 | 0.00 | 0.00 | 0.00 | 0.46 | 0.08 |
| multimodal | 2.54 | 4.24 | 5.64 | 3.51 | 2.31 | 5.25 | 3.92 |

**Table 2:** Proportions (in %) of taxa represented at the last ICNs.

| **Taxa** | **International congress for neuroethology** | | | | | |  |
| --- | --- | --- | --- | --- | --- | --- | --- |
|  | **2010** | **2012** | **2014** | **2016** | **2018** | **2022** | **mean** |
| Amoeba | 0.00 | 0.00 | 0.00 | 0.00 | 0.26 | 0.00 | 0.04 |
| Plant | 0.00 | 0.00 | 0.00 | 0.00 | 0.00 | 0.23 | 0.04 |
| Cnidaria | 0.00 | 0.00 | 0.21 | 0.00 | 0.26 | 0.46 | 0.15 |
| Annelid | 1.37 | 1.12 | 1.04 | 0.27 | 0.77 | 0.68 | 0.88 |
| Molluscs | 4.31 | 4.91 | 4.80 | 2.70 | 3.59 | 2.28 | 3.77 |
| Onychophora/Tardigrade | 0.00 | 0.00 | 0.00 | 0.00 | 0.26 | 0.23 | 0.08 |
| Nematode | 0.98 | 0.22 | 2.30 | 0.81 | 1.03 | 0.91 | 1.04 |
| Myriapoda | 0.00 | 0.00 | 0.00 | 0.00 | 0.26 | 0.00 | 0.04 |
| Chelicerata | 1.57 | 1.12 | 1.25 | 0.81 | 2.31 | 1.83 | 1.48 |
| Crustacean | 3.72 | 4.46 | 5.01 | 4.86 | 7.18 | 4.11 | 4.89 |
| Insects | 42.27 | 36.38 | 39.04 | 33.78 | 34.36 | 37.67 | 37.25 |
| Echinodermata | 0.00 | 0.00 | 0.21 | 0.27 | 0.26 | 0.23 | 0.16 |
| Tunicate | 0.00 | 0.00 | 0.00 | 0.00 | 0.26 | 0.46 | 0.12 |
| Agnatha (Lamprey) | 0.39 | 0.00 | 0.21 | 0.00 | 0.00 | 0.00 | 0.10 |
| Fish | 14.48 | 18.97 | 8.98 | 18.92 | 16.92 | 20.09 | 16.39 |
| Amphibians | 5.68 | 7.14 | 3.13 | 3.51 | 3.85 | 2.97 | 4.38 |
| Reptiles | 1.76 | 1.34 | 1.04 | 1.35 | 3.33 | 0.46 | 1.55 |
| Birds | 6.65 | 9.60 | 12.94 | 11.35 | 7.69 | 6.85 | 9.18 |
| Mammals | 12.13 | 11.61 | 13.15 | 15.41 | 11.03 | 13.24 | 12.76 |
| comparative studies | 1.76 | 1.34 | 3.55 | 4.59 | 6.41 | 7.08 | 4.12 |

**Table 3:** Proportions (in %) of ICN contributions categorized into 55 research topics.

| **Research topics** | **International congress for neuroethology** | | | | | |  |
| --- | --- | --- | --- | --- | --- | --- | --- |
|  | **2010** | **2012** | **2014** | **2016** | **2018** | **2022** | **mean** |
| **communication** | 11.94 | 17.63 | 1.88 | 0.00 | 0.51 | 1.14 | 1.16 |
| **mating/courtship** | 9.39 | 9.38 | 0.00 | 0.54 | 1.28 | 3.42 | 1.08 |
| **odor tracking** | 0.00 | 2.23 | 0.00 | 0.00 | 0.00 | 1.14 | 0.33 |
| **predation** | 9.59 | 11.38 | 1.25 | 1.35 | 1.03 | 0.91 | 1.25 |
| **camouflage** | 0.59 | 0.89 | 1.88 | 2.43 | 1.28 | 1.37 | 1.49 |
| **fear** | 0.00 | 0.00 | 0.21 | 4.05 | 1.79 | 0.91 | 1.97 |
| **pain** | 0.78 | 0.67 | 4.18 | 2.43 | 3.59 | 1.83 | 3.18 |
| **aggression** | 3.13 | 2.68 | 17.33 | 13.78 | 12.56 | 12.10 | 14.22 |
| **adaptations to dim light** | 2.74 | 3.35 | 10.02 | 5.68 | 3.59 | 4.57 | 7.10 |
| **spatial orientation** | 12.72 | 15.40 | 1.04 | 0.27 | 1.79 | 0.91 | 0.70 |
| **electrolocation** | 2.15 | 2.68 | 15.45 | 14.86 | 9.23 | 11.87 | 11.52 |
| **echolocation** | 3.72 | 3.35 | 18.37 | 15.14 | 15.64 | 20.78 | 16.34 |
| **foraging** | 2.15 | 0.45 | 3.97 | 1.89 | 2.56 | 2.51 | 3.06 |
| **memory** | 10.57 | 7.14 | 1.46 | 1.62 | 1.54 | 1.60 | 1.54 |
| **social learning** | 0.00 | 0.67 | 0.42 | 0.00 | 0.26 | 0.46 | 0.45 |
| **hormones (pheromones)** | 5.87 | 4.24 | 4.38 | 4.59 | 2.56 | 4.57 | 3.12 |
| **cognition** | 0.78 | 0.22 | 2.30 | 2.97 | 2.31 | 4.11 | 2.92 |
| **attention** | 0.59 | 1.56 | 6.68 | 8.92 | 7.69 | 5.71 | 8.33 |
| **decision making** | 0.78 | 2.68 | 2.68 | 1.89 | 3.59 | 2.97 | 3.27 |
| **numerosity** | 0.20 | 0.00 | 2.46 | 3.51 | 2.56 | 2.74 | 2.46 |
| **hibernation** | 0.00 | 0.00 | 2.68 | 2.16 | 3.33 | 1.60 | 2.44 |
| **stress** | 0.20 | 0.00 | 3.35 | 3.78 | 5.90 | 1.83 | 3.49 |
| **parental care** | 0.00 | 0.00 | 1.56 | 3.78 | 2.31 | 2.97 | 2.34 |
| **aging** | 0.39 | 0.45 | 3.35 | 2.97 | 1.28 | 1.83 | 2.12 |
| **flight control** | 4.89 | 3.57 | 3.35 | 3.51 | 1.54 | 2.97 | 2.63 |
| **gravity sensing** | 0.39 | 0.22 | 8.93 | 7.30 | 4.10 | 5.94 | 6.06 |
| **proprioception** | 1.37 | 0.67 | 0.00 | 0.54 | 0.26 | 0.91 | 0.32 |
| **symbiosis** | 0.20 | 0.00 | 0.22 | 0.00 | 0.00 | 0.00 | 0.18 |
| **parasites/diseases** | 0.39 | 1.34 | 2.68 | 3.24 | 6.41 | 3.20 | 3.52 |
| **plasticity** | 0.39 | 2.23 | 0.00 | 1.62 | 0.00 | 1.37 | 0.70 |
| **nerve regeneration** | 0.20 | 0.00 | 1.12 | 4.59 | 1.03 | 0.68 | 1.67 |
| **brain size (allometry)** | 0.78 | 0.45 | 0.22 | 0.27 | 0.51 | 0.00 | 0.20 |
| **lateralization** | 0.39 | 0.00 | 1.79 | 0.54 | 0.77 | 2.97 | 1.30 |
| **binocularity** | 0.20 | 0.00 | 0.00 | 0.00 | 0.26 | 0.91 | 0.23 |
| **polarization vision** | 1.76 | 3.13 | 0.45 | 0.27 | 0.00 | 0.46 | 0.26 |
| **color vision** | 2.94 | 2.68 | 0.45 | 0.81 | 1.28 | 1.14 | 0.97 |
| **impulse control** | 0.39 | 0.45 | 0.00 | 0.27 | 0.77 | 0.91 | 0.39 |
| **state dependence** | 2.15 | 2.23 | 0.22 | 0.00 | 0.51 | 0.23 | 0.40 |
| **biological clocks** | 0.59 | 2.68 | 1.56 | 0.27 | 0.77 | 2.05 | 1.57 |
| **social behavior, e.g. eusocial** | 2.54 | 0.89 | 0.89 | 0.54 | 1.54 | 1.37 | 1.14 |
| **internal or external noise** | 2.94 | 4.46 | 1.12 | 0.54 | 0.77 | 1.14 | 0.93 |
| **stimulus specific adaptation** | 1.37 | 0.22 | 0.00 | 0.00 | 0.00 | 0.00 | 0.10 |
| **sound localization** | 0.78 | 2.23 | 0.67 | 0.54 | 1.03 | 1.37 | 0.89 |
| **sleep** | 0.20 | 0.00 | 0.45 | 0.27 | 0.26 | 0.46 | 0.27 |
| **recognition of conspecifics** | 0.39 | 1.56 | 0.89 | 1.35 | 1.28 | 0.91 | 1.37 |
| **sexual dimorphism** | 1.37 | 1.56 | 0.89 | 0.27 | 0.26 | 0.23 | 0.48 |
| **motor efferences** | 0.39 | 0.45 | 1.34 | 0.00 | 1.03 | 0.91 | 0.79 |
| **collective behavior** | 0.78 | 0.45 | 0.22 | 0.27 | 0.00 | 0.00 | 0.22 |
| **individual variability** | 0.78 | 0.45 | 1.56 | 7.57 | 1.79 | 5.94 | 2.98 |
| **natural stimuli** | 0.20 | 1.56 | 0.22 | 0.81 | 0.00 | 0.46 | 0.36 |
| **tracking techniques** | 1.96 | 0.00 | 0.45 | 0.27 | 0.51 | 0.46 | 0.28 |
| **oudoor recordings** | 0.39 | 0.00 | 2.23 | 2.16 | 0.51 | 1.14 | 1.01 |
| **virtual reality** | 1.17 | 1.34 | 1.34 | 2.16 | 0.77 | 0.91 | 0.90 |
| **robotics** | 2.74 | 2.01 | 0.45 | 0.81 | 2.82 | 2.97 | 1.50 |
| **automated quantifications** | 1.57 | 2.23 | 0.00 | 0.00 | 0.26 | 0.46 | 0.12 |
